# Supplementary figures and images for: Correlations between behavior and hormone concentrations or gut microbiome imply that domestic cats (Felis silvestris catus) living in a group are not like ‘groupmates’
Source: PLoS One. 2022 Jul 27;17(7):e0269589. doi: 10.1371/journal.pone.0269589 (PMC9328509; doi:10.1371/journal.pone.0269589)

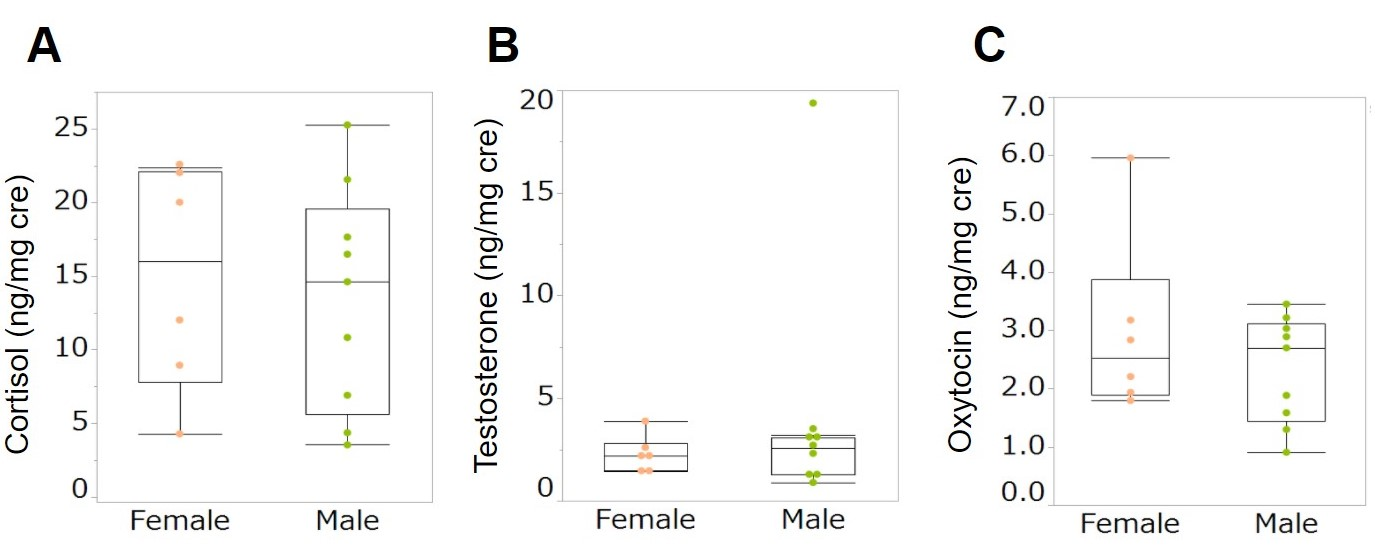

Supplement: S1 Fig — S1A Fig indicates cortisol, S1B indicates testosterone, and S1C indicates oxytocin. Each point shows average hormone concentration for each individual. (TIF) [file pone.0269589.s001.tif]

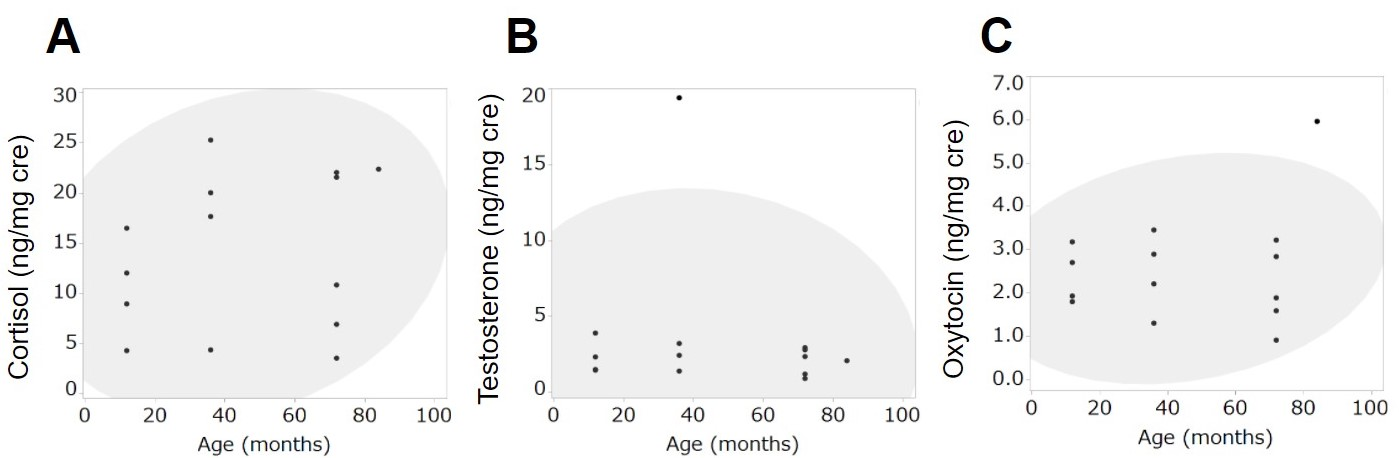

Supplement: S2 Fig — S2A Fig indicates cortisol, S2B indicates testosterone, and S2C indicates oxytocin. Each point shows average hormone concentration for each individual. The gray circle is a 95% confidence ellipse. (TIF) [file pone.0269589.s002.tif]

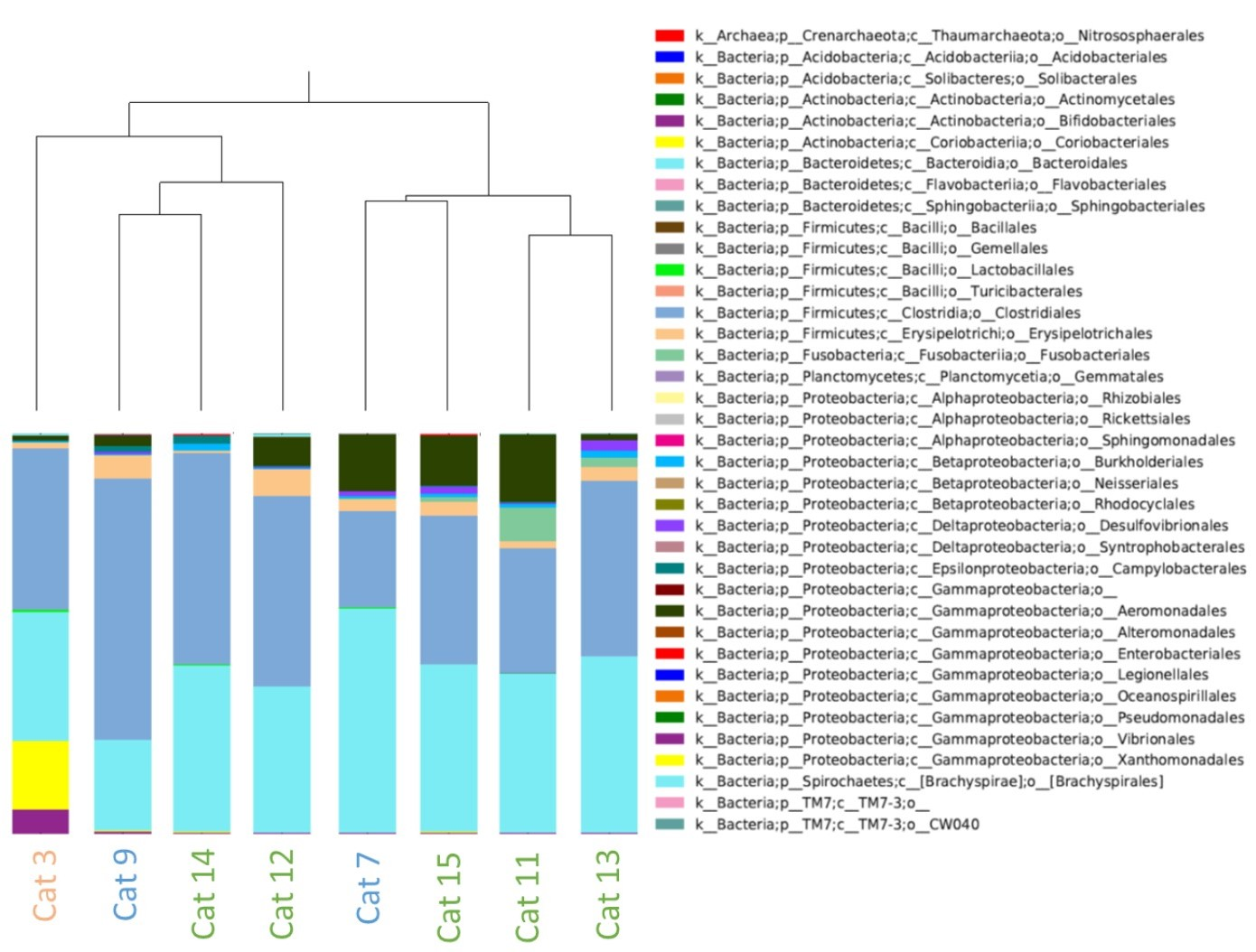

Supplement: S3 Fig — The hierarchical clusters are shown based on the percentages of gut microbes at the genus level. The colors of each cat are color-coded for each group (Orange: Group 1, Blue: Group 2, Green: Group 3). (TIF) [file pone.0269589.s003.tif]
