# Supplementary material for: Correlations between behavior and hormone concentrations or gut microbiome imply that domestic cats (Felis silvestris catus) living in a group are not like ‘groupmates’
Source: PLoS One. 2022 Jul 27;17(7):e0269589. doi: 10.1371/journal.pone.0269589 (PMC9328509; doi:10.1371/journal.pone.0269589)
Supplement: Attachment — Submitted filename: Response to reviewers.docx [file pone.0269589.s016.docx]

24.01.2022

Chun Wie Chong

Academic Editor

*PLOS ONE*

Dear Dr. Chun Wie Chong

Thank you for inviting us to submit a revised draft of our manuscript (PONE-D-21-34568) titled “Correlation between behavior and hormone or gut microbiome implies that domestic cats (*Felis silvestris catus*) living in a group are not like ‘groupmates’”. We appreciate the time and effort you and each of the reviewers have dedicated to providing insightful feedback on how to strengthen our paper. Thus, it is with great pleasure that we resubmit our article for further consideration. We have incorporated changes that reflect the detailed suggestions you have graciously provided. We hope that our edits and the responses we provide below satisfactorily address all issues and concerns you and the reviewers have noted. Please find our point-by-point responses to the reviewers' comments below.

I am looking forward to hearing from you.

Sincerely,

Hikari Koyasu

Human-Animal Interaction and Reciprocity Laboratory,

Azabu University

### Reviewer #1:

*The manuscript addresses an interesting relationship between behaviour, hormones and gut microbiota of group-living cats. However, the methodology and statistics used in this study is not well-explained and lack sufficient detail to evaluate the relevance of the findings.*

Reply: We thank the reviewer for all the detailed comments and suggestions. We found them very useful as we approached our revision.

*First, the analysis of gut microbiota section must have a detailed information about the laboratory and bioinformatic processing. Even if the processing was done by a company all the information about the processing steps need to be addressed. There is no information about extraction method, PCR settings, library building, primers, sequencing strategy, if biological or technical replicates were used, how many blanks and what kind were used… Moreover, the faecal samples were collected using two different method? Was this controlled in the analysis?*

Reply: We have added detailed information about the bioinformatic processing of gut microbiomes (lines 146-175).

In addition, as mentioned by the reviewer, the fecal samples were collected using two methods, and it was best to collect them in one consistent way. The sampling method for the first group was for 16S rRNA analysis, in addition to the storage of bio-active microbiomes (glycerol stock). However, the application of this method would have involved high efforts and exceeded our storage space, so that we changed the collection method. The time of the sampling did not change the composition of the microbiome as long as we collected the part of the feces that was not exposed to the air near the center (Sinha et al. 2016). Moreover, the use of the swab did not change the composition of the microbiome (Sinha et al. 2016). Therefore, targeting a small number of bacteria in the fecal microbiome that are sensitive to oxygen may not eliminate the effect of differences in sampling methods, but by comparison of the overall structure, we determined that there were small effects on the analysis.

*Second, it is not sufficient to write that the “data were processed using QIMME”, the author should include detailed information about how the sequences were analysed (e.g. demultiplexing, primer removal, filtering, denoising, contaminant removal…). In addition, the authors should make the bioinformatic pipeline available.*

Reply: We have added bioinformatic information and information about the analysis of the gut microbiome（lines 176-191).

*Third, it is not clear to me how the faecal samples were collected. In the experimental setting section, the authors say that 8 faecal samples were collected, but from which individuals? Was it possible to identify who owned each stool sample? Otherwise, how was it possible to correlate a specific microbial community with a specific behaviour? And finally, have you considered in the analysis that some faecal samples belong to cats that share a room (5 rooms and 8 faecal samples)? Therefore, some samples are not independent. How was this controlled in the statistical analysis?*

Reply: We collected the fecal samples from different individuals only when we observed which cats excreted the feces. Moreover, the experiment was conducted for one group at a time, and the three groups stayed in the same room. In addition, all cats were eating the same food. We have reduced the influence of the similarity of the gut microbiome through the room environment and food as much as possible. We have rewritten the method description on the fecal collection and experimental setting (lines 146, 96-98), and individuals for which fecal samples were collected are shown in Table S1 to address the reviewer’s comment. We hope that the revised section is now clarified.

However, it was not possible to completely eliminate the effects of the environment because each group was in the specific environment at different times. Since we were only able to collect samples from one cat in the first group and two cats in the second group, it was difficult to take into account that there were different groups in this study. Therefore, we have added to the discussion that it is necessary to examine the gut microbiome of more cats living in the same room at the same time (lines 325-329).

*Fourth, since the relevant correlations (and p-values) are already included in the text and the tables do not provide any additional information, all the tables should be moved to supplementary material.*

Reply: We thank the reviewer for the suggestion. All tables have been moved to the supplementary material.

*Lastly, the discussion part should be re-organized to clarify the findings of this study and its consequences.*

Reply: We have rewritten the discussion to clarify the findings and their consequences.

### Reviewer #2:

*This study provides insight between behavior of shelter cats correlated to hormonal and gut microbiome profiling. The conclusions obtained were valuable as the findings shed light on the hormonal status of originally solitary or group-living animals. It is hopeful that the conclusions drawn from this study can be an example to help behaviorists incorporate more measurable parameters which will reduce subjectivity when relying only on visual observations. Despite the gender-neutral assumption, it might be worthwhile to have gender-specific groupings to further understand its impact on behavior for future studies, in addition to increased sample size and longer duration. The methods used were sound and results obtained provided clarity on the hypotheses.*

Reply: We thank the reviewer for the useful comments. We agree on the importance of conducting further research with gender-specific groupings. The differences in frequency of behaviors that occur within males and females, and between males and females should be investigated by addition of further cats. We have added this information to the discussion (lines 351-352).

### Reviewer #3:

*1. Interesting. The authors addressed notable correlation between hormones and behaviours. While mounting studies had established the correlation between gut microbiome and behaviours, the present study lacking clarity in term of study design for determining the gut microbiota constituents. Microbiota composition varies inter-individually, and effected by various environmental factors. The recent study only sampled the cats stool at one point of time. It is suggested to sample the stool similarly like urine, and sequence the stool concurrently i.e. time series analysis for the gut microbiome.*

We thank the reviewer for raising this important point. For this gut microbiome analysis, we used feces collected as close to the end of the two-week experimental period as possible. Although only suggestive, this shows an association between the frequency of interactions among individuals during two weeks and the similarity of the gut microbiome as close to the end of that period as possible.

As pointed out by the reviewer, microbiome composition varies inter-individually and is affected by various environmental factors, including food, the presence of other individuals, interaction with others, and so on. Sampling at various time points and examination of the relationship between changes of gut microbiomes in the time series and the frequency of contact with other individuals would be required. In addition, the possibility of transmission of bacteria through a shared environment has not been completely eliminated, and this is an issue that needs to be investigated as a future task. We have added these concerns to the discussion (lines 325-329).

*2. The grouping strategy was vaguely described.*

Reply: As for the grouping, it was completely random. Since the number of males and females was not consistent, we have added to the discussion that it will be necessary to conduct experiments in groups with a consistent number of males and females, as well as in groups with only males or only females to understand the adaptive significance of group formation (lines 351-352).

*3. While the hormonal analyses were conducted for 50 plus samples, the microbiome analysis was conducted only on 8 cats subjects, with Group 1 only represented by 1 subjects.*

Reply: This was a matter of concern to us. We could not collect many feces from the cats, but this was due to the fact that we only collected feces when we could see who had defecated at the time of defecation, and the cats often defecated when humans were not around. We have added that future analyses of time series with larger sample sizes are needed (lines 325-329).

*4. Sample preparation (microbial genomics library preparation) was vaguely described.*

Reply: We thank the reviewer for pointing out this point. We have added detailed information about the sample preparation, such as microbial genomics library preparation (lines 156-175).

*5. Microbial genomics analysis was vaguely described.*

Reply: We have added detailed information about the microbial genomics analysis (lines 176-191).

*6. The raw sequencing data were not publicly available. Data availability. We recommend that all data and related meta-data underlying the findings reported in a submitted manuscript will be deposited in an appropriate public repository (GenBank, European Nucleotide Archives, National Center for Biotechnology and Information, National Bioscience Data Center, DNA data bank of Japan).*

Reply: We thank the reviewer for the suggestion. The data has been deposited in the DNA Data Bank of Japan (https://www.ddbj.nig.ac.jp/index.html: accession number PRJDB12856).

*7. Is there any specific reason to opt for UniFrac distance?*

Reply: We opted for the UniFrac distance because it enables us to compare the differences in the overall microbiome between two samples while considering phylogeny. The UniFrac distance takes into account the distance between bacterial phylogeny, which enables principal coordinate analysis.

*8. The manuscript was written in standard English, with minor grammatical errors.*

Reply: Our manuscript was again corrected by native speakers.

*9. Data presentation using PCoA were OK, but making individual comparison for microbiome analysis was vary confusing. I would suggest for the authors to conduct group comparison for the microbiome analysis. In fact, inter group correlation between behaviours also can be conducted*

Reply: We thank the reviewer for the suggestion. We agree on the relevance of conducting an intergroup comparison of the gut microbiome, but this was not possible due to the insufficient number of cases (only one sample in the first group and two samples in the second group). When we show that different groups have different gut microbiome compositions , we cannot exclude the possibility of similarity from the shared environment. Therefore, we examined the relationship between the frequency of contact between individuals and the degree of similarity of their gut microbiome. To avoid any confusion, we have reorganized the results section (lines 243-250).
