# Supplementary material for: Correlations between behavior and hormone concentrations or gut microbiome imply that domestic cats (Felis silvestris catus) living in a group are not like ‘groupmates’
Source: PLoS One. 2022 Jul 27;17(7):e0269589. doi: 10.1371/journal.pone.0269589 (PMC9328509; doi:10.1371/journal.pone.0269589)
Supplement: Attachment — Submitted filename: Response_to_reviewers_v3_final.docx [file pone.0269589.s017.docx]

Reviewer #1:

I believe that the ms has improved significantly, however, I still have some doubts about the reliability of the results coming from the analysis of microbial data. The authors do not mention the use of controls and, in addition, they did not perform any filtering steps to minimise the introduction of non-real reads. Therefore, the reliability of these results is highly compromised.

**Reply: We appreciate the reviewer for all the detailed comments and suggestions. We found them very useful as we undertook our revision.**

**Instead of using the PhiX control, we have shifted the reading frame by adding 0-5 random N in front of the primer sequence. As for the non-real reads that the referee pointed out, it seems to be due to the following three issues: low quality reads, chimeras, and non-bacterial reads. We removed sequences with a quality value < 20, and all sequences were checked for chimeras, and those not determined to be chimeric were extracted and used for subsequent analyses. The information has been provided in the manuscript. For the processing of non-bacteria reads, non-bacteria reads are removed after annotation. This has been added to the methods: In addition, if there were non-bacterial reads, they were removed (lines 197-198).**

With regard to statistical analysis, more than one urine sample was collected from an individual to assess hormone levels, so the samples are not independent. How did you statistically analyse this dependency between the samples?

**Reply: Thank you for pointing this out. When we checked the factors affecting all urinary hormones using a generalized linear mixed model, we found that these hormone samples were not affected by date and that individual effects were significant. Therefore, the average of the urine hormone concentrations for each individual was used as the hormone baseline for each individual. We have added an additional explanation for the urinary hormone data: The average of the urine hormone concentrations for each individual was used as the hormone baseline for each individual (lines 121-122).**

Additionally, I think the authors should also modify the discussion so that the correlation between hormone levels and behaviours is easier to understand. For example: why is oxytocin in one subsection and cortisol and testosterone in another subsection? Also, at the end of the cortisol and testosterone section you mention oxytocin. So it is necessary to clarify the subsections or if they prefer to merge them all into one. It may be easier for the reader to understand the correlation of hormones and behaviour if the authors explain them all together. Since the discussion is mainly based on the correlation of hormones and affiliative contact with peers (tolerance towards other individuals), I would recommend discussing the results according to behaviour. What are the hormone levels related to affiliative behaviour? Otherwise it is very repetitive.

**Reply: We appreciate the reviewer's concerns on this point. As the reviewer pointed out, the topic of oxytocin in the cortisol/testosterone section could cause confusion; therefore, the section has been reorganized. Since the study is based on the hypothesis that hormones modify cat behaviors, discussions have been made based on hormones, and we would like to maintain this approach.**

L28-29 Is it only the environment that shapes it? Genes have no effect? I would say that environment is one of the variables that affect behaviour. This sentence should be modified and more references added.

**Reply: As the reviewer points out, the environment is one of the factors that affect behavior. We have corrected the relevant sentence: During evolution, ecological factors, including food availability, influence the development of group and solitary living behavior in animals [1] (lines 27-28).**

L33: What do you mean domesticated cats? Animals that live with humans as pets? or feral cats that live in cities. Please clarify it. I don't think cats LIVE in high densities, but rather in large groups. We can find high densities of cats in cities and towns, but not all of these cats live together as a group.

**Reply: Domesticated cat in this context refers to the domesticated cat as a species, which includes both cats living in homes with humans and feral cats. While many species of felids live alone and have exclusive territories, domestic cat species are able to live at relatively high densities in specific spaces. The definition of a group varies. Therefore, in the present context, we do not use the word 'group' but 'high density' because it is unclear what kind of group the cats form.**

L46-47: Add References

**Reply: We have added references.**

**e.g.,**

**M Stöwe; T Bugnyar; C Schloegl; B Heinrich; K Kotrschal; E Möstl. Corticosterone excretion patterns and affiliative behavior over development in ravens (Corvus corax). Horm Behav 53, 208–216 (2008)**

**FB de Waal; F Aureli; PG Judge. Coping with crowding. Sci Am 282, 76–81 (2000)**

**DA Gust; TP Gordon; MK Hambright; ME Wilson. Relationship between social factors and pituitary-adrenocortical activity in female rhesus monkeys (Macaca mulatta). Horm Behav 27, 318–331 (1993)**

**MN Barbosa; MT da S Mota. Behavioral and hormonal response of common marmosets, Callithrix jacchus, to two environmental conditions. Primates 50, 253–260 (2009)**

**C Crockford; RM Wittig; K Langergraber; TE Ziegler; K Zuberbuhler; T Deschner. Urinary oxytocin and social bonding in related and unrelated wild chimpanzees. Proc Biol Sci 280, 20122765 (2013)**

L52. Delete this sentence, you already mentioned it in the previous sentence.

**Reply: Thank you for the suggestion. The previous sentence was about various species, and the relevant sentence described cats. We have added an explanation that these describe a variety of species or cats.**

L79: did not measure the AMOUNT of specific intestinal microbiota, but rather the composition

**Reply: The reviewer’s comment is correct. We have corrected the sentence: 3. there is a relationship between the composition of gut microbiomes and hormone concentrations in an individual’s body (lines 80-81)**

L81-82: It is not clear to me what “solitary mammal within the same species” refers to.

**Reply: ‘Solitary mammal within the same species’ means that ‘role of oxytocin in conspecific social behaviors in a solitary mammal.’ To clarify, we have edited the sentence (lines 83).**

L85: Indicate the name of the shelter and the locality

**Reply: We have added the name of the shelter and the locality (line 87).**

L86: Have these animals received any health treatment, such as antibiotics?

**Reply: The cats in the experiment were not treated with antibiotics or other treatments.**

L113: You already mentioned how many samples you collected in L96. You only need to write it once.

**Reply: Thank you for pointing this out. We deleted the sentence with the same information in the experimental setting.**

L123-124: Explain why the concentrations for creatinine correction were adjusted and how it was done.

**Reply: All urinary hormone concentrations vary greatly, depending on the concentration of urine. Since the daily excretion of creatinine is constant (Shaffer PA, 1908), the hormone concentration considering the concentration of urine can be calculated based on the amount of creatinine in that urine (Munro CJ et al., 1991).**

**Shaffer PA., The excretion of creatin and creatinine in health and disease., American Journal of Physiology, 1908;10:1-10.**

**Munro CJ, Stabenfeldt GH, Cragun JR, Addiego LA, Overstreet JW, Lasley BL., Relationship of serum estradiol and progesterone concentrations to the excretion profiles of their major urinary metabolites as measured by enzyme immunoassay and radioimmunoassay., Clinical Chemistry, 1991;37:838-844.**

L117-125-134: There are no references on the measurement of cortisol, testosterone… Explain how these protocols were established and add references if based on previously published protocols.

**Reply: These protocols for hormone assays were based on previous studies. Related references have been added.**

L165: Add reference for primers.

**Reply: We have added an appropriate reference for the primers.**

**Klindworth, A., Pruesse, E., Schweer, T., Peplies, J., Quast, C., Horn, M., & Glöckner, F. O. (2013). Evaluation of general 16S ribosomal RNA gene PCR primers for classical and next-generation sequencing-based diversity studies. Nucleic acids research, 41(1), e1-e1.**

L178 - Fastx tool kit needs a reference

**Reply: We have added an appropriate reference.**

**A Gordon; GJ Hannon; Others. Fastx-toolkit. FASTQ/A short-reads preprocessing tools (unpublished) http://hannonlab cshl edu/fastx_toolkit 5 (2010)**

L179: Explain the function and the package used to perform quality control. Add the corresponding references.

**Reply: Fragment Analyzer and dsDNA 915 Reagent Kit (Advanced Analytical Technologies, Inc.) were used to check the quality of the libraries. We have added a relevant sentence and reference: The quality of libraries was evaluated using a Fragment Analyzer and a dsDNA 915 Reagent Kit (Advanced Analytical Technologies, Inc., USA).**

**N Susai; T Kuroita; K Kuronuma; T Yoshioka. Analysis of the gut microbiome to validate a mouse model of pellagra. Bioscience of Microbiota, Food and Health advpub, 2021–2059 (2022)**

L181:Add references for FLASH and uchimeras

**Reply: We have added the relevant references.**

**FLASH: T Magoč; SL Salzberg. FLASH: fast length adjustment of short reads to improve genome assemblies. Bioinformatics 27, 2957–2963 (2011)**

**UCHIME: RC Edgar; BJ Haas; JC Clemente; C Quince; R Knight. UCHIME improves sensitivity and speed of chimera detection. Bioinformatics 27, 2194–2200 (2011)**

L184-185: I guess you removed the chimeric sequences and then grouped them together and finally added the taxonomy. But this is not clear to me in the text, you should explain each step well and add the function used and its references.

**Reply: We have changed the sentences: The sequences that passed all the filtering were checked for chimeras using the uchime algorithm of usearch[61]. The reads that were not determined to be chimeras were used in subsequent analyses. In addition, if there were non-bacterial reads, they were removed. The creation of OTUs and phylogenetic estimation were performed using QIIME workflow scripts with no reference and all parameters set to default conditions. The number of reads used in the QIIME analysis was 224,878 (28,110 ± 1764) (lines 195-200).**

L188-190: Just mention it in the statistical analysis section

**Reply: We have removed the sentence and moved it to the statistical analysis section.**

L280: Add examples considered as affiliative behaviours in your study

**Reply: We considered allo-grooming, entering bed and sharing bed as affiliative behaviors in the present study. Since the affiliative behavior in the previous studies is mainly allo-grooming, we have described it.**

L287-289: It is not clear what additional information you provide from the previous sentence

**Reply: We have revised this part for enhanced clarity: The experimental period of two weeks may be too short to enable the formation of a tight relationship group and observing them for a longer period could result in a change in their relationships. Although their relationships were possibly established since the cats who participated in the experiment had been living in the same room at the shelter before the experiment, investigations over a longer duration could offer greater insights on group-formation in cats (lines 290-294).**

L300: Are cortisol and testosterone levels correlated? Are both related to fearlessness?

**Reply: Yes, there was a positive correlation between cortisol and testosterone concentrations. Yes, both are related to fearlessness.**

L303: Add a reference

**Reply: This is a suggestion based on the results of the present study and there is no previous study reporting such results. A comparison should be made to examine whether testosterone is associated with any of the behaviors in castrated and uncastrated cats. To clarify, we have clearly stated that this sentence is derived from the results of the present study.**

L304-307: It is not clear which results are from this study and which are from other studies. Please clarify this and add references when referring to other studies.

**Reply: Same as in the previous point, we have made them clearer.**

Reviewer #3:

Although the author has made corrections according to the recommendations given, I still see weaknesses in the study design for this study. With an unbalanced sample size for each group of cats, it is too difficult to draw conclusions. For example, the composition of the bacteria identified in this study is not discussed in depth. So it is too far -fetched for the authors to draw comparative conclusions involving the gut microbiome. Finally, I can't see the crosstalk between hormones and the composition of the cat's gut microbiota. This makes this study not strong.

**Reply: We appreciate you pointing this out. The fecal sample size in this study is a a major source of concern. Although it would have been ideal to collect feces from all individuals, limited feces were collected from all individuals within the two weeks. Some of the cats were afraid of people and would not defecate when people were present, because they were from a shelter. Therefore, data are limited due to the small sample size, and the results of the present study only suggest that there is a relationship between gut microbiome and behaviors and hormones. Nevertheless, the preliminary results could guide and facilitate future studies on cat microbiomes and the correlation with behavior.**

Reviewer #4:

Dear authors,

I appreciate the edits already completed by the authors from reviewer 1-3. However, this publication is still not at an acceptable standard for publication. The premise of the study is excellent, and it is a very interesting paper. However, authors must complete major edits, as I have begun to outline below:

**Reply: Thank you very much for providing such critical comments. In the following sections, you will find our responses to each of your points and suggestions. We are grateful for the time and energy you expended in reviewing this manuscript.**

The use of colloquial English ('we', "attempted to", "close to significant" etc.) must be corrected. Abbreviations need only be explained once when first used.

**Reply: We have revised the entire manuscript accordingly, with checks by native speakers.**

References must be added to statements on existing literature in the introduction, and species specific references included as opposed to blanket statements.

**Reply: We have added species-specific references (cats/other felids) and noted the associated species.**

**M Franchini; A Prandi; S Filacorda; EN Pezzin; Y Fanin; A Comin. Cortisol in hair: a comparison between wild and feral cats in the north-eastern Alps. Eur J Wildl Res 65, 90 (2019)**

**H Finkler; J Terkel. Cortisol levels and aggression in neutered and intact free-roaming female cats living in urban social groups. Physiol Behav 99, 343–347 (2010)**

**G Genaro; CR Franci. Cortisol influence on testicular testosterone secretion in domestic cat: An in vitro study. Pesqui Vet Bras 30, 887–890 (2010)**

The analytical methods used to assess hormone concentrations must be referenced. Standard units must be used (e.g. µl not ul), kits and reagents must have the manufacturing company and country of origin specified in brackets.

**Reply: We have added the appropriate references. In addition, standard units have been corrected and countries of origin for kits and reagents added.**

Supplementary tables provide little extra information, where are the notes indicating what rs denotes? Why is there a ? in front of these rs scores? Consistency must be addressed e.g. "p-value" and "p". Figure's require legends which thoroughly describe the research presented. In an example, Figure 1A-C, 62 samples were analysed for hormone concentrations, yet there are 14 data points? What do the grey circle denote?

**Reply: Thank you for pointing this out. The ‘rs’ represent correlation coefficients. The text in the supplemental table was garbled and a ‘?’ showed a negative value. We have corrected these points. In addition, explanations have been added to describe the result presented comprehensively.**

Also authors must note that they are analysing the faecal microbiome, not the gastrointestinal microbiome.

**Reply: Thank you for pointing this out. We have added the sentence: Most mammalian gut microbiome studies have focused on fecal microbiota, but it is unclear how well fecal samples reflect the microbiota of the intestinal region, and these should also be examined (lines 352-354).**

Additionally, here are some specific edits I began writing:

Line 46: Please provide references for this statement

**Reply: We have added the relevant references.**

**e.g.,**

**M Stöwe; T Bugnyar; C Schloegl; B Heinrich; K Kotrschal; E Möstl. Corticosterone excretion patterns and affiliative behavior over development in ravens (Corvus corax). Horm Behav 53, 208–216 (2008)**

**FB de Waal; F Aureli; PG Judge. Coping with crowding. Sci Am 282, 76–81 (2000)**

**DA Gust; TP Gordon; MK Hambright; ME Wilson. Relationship between social factors and pituitary-adrenocortical activity in female rhesus monkeys (Macaca mulatta). Horm Behav 27, 318–331 (1993)**

**MN Barbosa; MT da S Mota. Behavioral and hormonal response of common marmosets, Callithrix jacchus, to two environmental conditions. Primates 50, 253–260 (2009)**

**C Crockford; RM Wittig; K Langergraber; TE Ziegler; K Zuberbuhler; T Deschner. Urinary oxytocin and social bonding in related and unrelated wild chimpanzees. Proc Biol Sci 280, 20122765 (2013)**

Line 59-61: Usually this is just called the gut-brain axis (GBA). References must be included at the end of this sentence.

**Reply: We have added the relevant references.**

**JF Cryan; KJ O’Riordan; CSM Cowan; KV Sandhu; TFS Bastiaanssen; M Boehme; MG Codagnone; S Cussotto; C Fulling; AV Golubeva; KE Guzzetta; M Jaggar; CM Long-Smith; JM Lyte; JA Martin; A Molinero-Perez; G Moloney; E Morelli; E Morillas; R O’Connor; JS Cruz-Pereira; VL Peterson; K Rea; NL Ritz; E Sherwin; S Spichak; EM Teichman; M van de Wouw; AP Ventura-Silva; SE Wallace-Fitzsimons; N Hyland; G Clarke; TG Dinan. The Microbiota-Gut-Brain Axis. Physiol Rev 99, 1877–2013 (2019)**

Line 62-65: Please state which model (human, rodent, in vitro) these studies were conducted in, as there is currently no studies which have assessed this in domestic cats.

**Reply: Thank you for the suggestion. For enhanced clarity, we have added the models in the studies.**

Line 67-72: Again, please state which species you are referring too. These statements form a more compelling argument for the group-living of domestic cats, if they are referring to research conducted in prides of lions as opposed to a school of dolphins.

**Reply: These references were mainly about insects and rodents. We have identified the species examined in the studies.**

Line 85: Please add a statement declaring where the cats were housed during the study. E.g. Adult domestic shorthair cats were housed at the XX facility/shelter, Japan for the duration of the study.

**Reply: All cats were housed in one room at Azabu University during the experiment. We have added information on where the animals were maintained during the experiment: All cats were housed in one room at Azabu University during the experiment.(lines 89-90).**

Line 89: two weeks

**Reply: We have corrected (line 93).**

Line 90: litter tray?

**Reply: Yes, those were litter trays. We have changed ‘toilets’ to ‘litter trays (line 94).’**

Line 97: As diet is such an important factor for the microbiome, it would be great to have the dietary information here. Even if only to add in a sentence such as: “Cats were fed a complete and balanced commercially available kibble/can diet”

**Reply: We have added a sentence about diet: All cats were fed a complete and balanced commercially available kibble diet (lines 100-101).**

Line 113: For future studies, collect urine from individuals and note which cat produced it. This is a far more accurate way of collecting data, rather than expressing the sample as a number per individuals.

**Reply: Thank you for your comment. In the present study, we recorded which cats excreted and the time. A minimum 1 - maximum 9 urine samples were collected per individual. We have added the sentence in the hormonal assay section: Sixty-three urine samples, i.e., 4.2 ± 2.4 samples/individual (A minimum of one and a maximum of nine urine samples were collected per individual) were collected during the entire observation period… (lines 116-117).**

Cortisol/Creatinine/Testosterone/Oxytocin Concentrations: Were these methods derived from existing published methods? If so, please insert references.

**Reply: We have added the relevant references.**

**T Nagasawa; M Ohta; H Uchiyama. The Urinary Hormonal State of Cats Associated With Social Interaction With Humans. Front Vet Sci 8, 680843 (2021)**

**K Uetake; A Goto; R Koyama; R Kikuchi; T Tanaka. Effects of single caging and cage size on behavior and stress level of domestic neutered cats housed in an animal shelter. Anim Sci J 84, 272–274 (2013)**

**K Carlstead; JL Brown; W Strawn. Behavioral and physiological correlates of stress in laboratory cats. Appl Anim Behav Sci 38, 143–158 (1993)**

**K Kojima; S Hguchi; N Tashiro. Changes in urinary cortisol associated with hypthalamically elicited restlessness. Stress Med 11, 61–65 (1995)**
